# Microporous hierarchically Zn-MOF as an efficient catalyst for the Hantzsch synthesis of polyhydroquinolines

**DOI:** 10.1038/s41598-022-05411-8

**Published:** 2022-01-27

**Authors:** Sayed Mohammad Ramish, Arash Ghorbani-Choghamarani, Masoud Mohammadi

**Affiliations:** 1 Department of Chemistry, Faculty of Science, Ilam University, P. O. BOX. 69315516, Ilam, Iran; 2 Department of Organic Chemistry, Faculty of Chemistry, Bu-Ali Sina University, 6517838683 Hamedan, Iran

**Keywords:** Catalysis, Environmental chemistry, Inorganic chemistry, Materials chemistry, Organic chemistry

## Abstract

A three-dimensional walnut-like Zn-based MOF microsphere system was designed and synthesized via hydrothermal reaction of zinc salt with 4,6-diamino-2-pyrimidinethiol as a tridentate ligand. Besides, Zn ions were coordinated to the functional groups of the ligand to give a novel Zn-MOF microsphere material. Afterward, the resultant material was thoroughly characterized using various analysis and physico-chemical methods; including, FT-IR, XRD, TGA, EDX, X-ray mapping, SEM, TEM, and BET analysis. The Zn-MOF microspheres were utilized in the Hantzsch reaction for a selective synthesis of asymmetric polyhydroquinolines, using various aromatic aldehydes. Our strategy aims at providing a controlled synthesis of hierarchically nanoporous Zn-MOF microspheres with a well-defined morphology, structure, and excellent catalytic properties. Besides, it would result in having a promising heterogeneous catalyst for a selective synthesis with good yields, short reaction time, a low limit of steric hindrance and electronic effects. Moreover, the heterogeneity of the catalyst is further tested with hot filtration and also the reusability results point.

## Introduction

Porous materials with large specific surface areas and other unique properties have gained increasing attention^1–3^. During the last two decades, it has been revealed that Metal–organic frameworks (MOFs) have extensive applications in drugs delivery, absorption and desorption of substances, biomedicines, and catalysis due to their desired morphology, high surface area, stability, active metal sites and controllable pore size^4–11^. In this sense, coordination polymer microspheres have drawn great attention for their potential applications in many fields especially in the catalytic synthesis of various organic and bioorganic molecules via organic transformations^12–15^. They can be regarded as a novel class of crystalline porous compounds formed by the strong bonding of metal ions or clusters to polytopic organic ligands, giving rise to extended networks^13,16^. Since traditional microspheres have some drawbacks, i.e. higher product costs because of expensive excipients, sophisticated equipment and stricter quality control^17,18^, the nanoporous microspheres with low density and high specific surface area have become a pragmatic manner to address the existing problems^19–22^.

Recently, some specific efforts have been made to design effective microsphere catalysts with external pores on the surface or internal pores in the core (which can be regarded as the main difference between porous microspheres and traditional microspheres), instead of using traditional microsphere catalysts based on expensive metals^1,12,23–27^. Besides, porosity is very significant in measuring the capacity-efficiency of the catalysts. Moreover, the applied porous microspheres have been based on the porous structure, i.e. the amount, diameter, the structure of the pores, etc., which can be controlled by chemical and physical techniques^28,29^. Among all of the transition metal-based catalysts used in organic transformations, Zinc has gained great attention due to its precious properties such as low cost, nontoxicity, good availability, and being ecofriendly. Therefore, many studies have shown the implementation of catalytic systems based on Zn using various linkers in organic reactions^30–35^.

The asymmetric reaction of an aldehyde, two components of β-keto esters, and a nitrogen donor is known as Hantzsch method which generates chiral dihydropyridines. Besides, they have been regarded as useful precursors to many compounds of biological and pharmaceutical interest, i.e. different natural products^36,37^. The application and synthetic methods for polyhydroquinolines synthesis have previously been reviewed^36^. When transition metal catalysts are present, polyhydroquinolines with the functionalized aromatic rings can be generated from this reaction^38–41^. Among various catalysts developed so far, the efficiency of the catalytic systems is generally limited by their low yield, use of volatile organic solvents, harsh reaction condition, aggregation tendency as well as poor mechanical properties, high catalyst loading, tedious workup procedure, and separation of catalyst residue from the reaction mixture^36,42^. Although this method is practically useful for the synthesis of interesting pharmaceutical products, its selectivity and substrate scope still need to be improved^36,43,44^. We propose to develop a novel selective heterogeneous catalytic system for the recognition of the polyhydroquinoline derivatives to facilitate the screening of the catalysts for this asymmetric reaction.

In this work, we studied the hydrothermal synthesis of novel Zn-based nanoporous microspheres, by the reaction of zinc nitrate hexahydrate salt with 4,6-Diamino-2-pyrimidinethiol containing amine, thiol, and pyrimidine functional groups. We also found out that Zn-MOFs can be used as a rapid catalyst to synthesize the polyhydroquinolines with excellent yields. It represents a new method for a clean and rapid synthesis of asymmetric dihydropyridines. Herein, these results are reported.

## Experimental

### Materials

All reagents and solvents were purchased from Merck and used without additional purification.

### Synthesis of nanoporous Zn-MOF microspheres

**Scheme 1:**
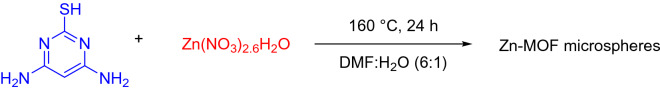
The synthesis of Zn-MOF microspheres.

To prepare Zn-MOF porous microspheres, 4,6-Diamino-2-pyrimidinethiol (1 mmol) was dissolved in water (2 mL) and, then, it was added to a solution of DMF (12 mL) containing 2 mmol of Zn(NO_3_)_2_.6H_2_O salt through 20 minutes of stirring at 80 °C until reaching a clear solution. Afterward, the white suspension was transferred into a Teflon lined autoclave reactor and heated at 160 °C for 24 h (Scheme 1), which was then cooled to the room temperature at a gradient of 40 °C per hour. Subsequently, the target Zn-MOF microsphere yellow powder was obtained after the sonication in ethyl acetate.

### General procedure for the catalytic synthesis of polyhydroquinolines

A mixture of the substituted aromatic aldehyde (1.0 mmol), ethyl acetoacetate (1 mmol) and dimedone (1 mmol) was stirred with Ammonium acetate – as a green NH_3_ source (1.2 mmol) – and Zn-MOF microspheres (8 mg) in PEG-400 (2 mL) for 70–180 min at 80 °C. Completion of the following reaction has been analyzed via TLC. Subsequent to cooling at room temperature, the catalyst was separated using simple filtration. Besides, the resultant mixture was extracted using Ethyl acetate and water derivation to gather unrefined polyhydroquinoline product, which was further purified through recrystallization in ethanol.

### Selected spectral data

#### Ethyl-4-(4-hydroxyphenyl)-2,7,7-trimethyl-5-oxo-1,4,5,6,7,8-hexahydroquinoline-3-carboxylate

^1^H NMR (500 MHz, DMSO-d6): δ (ppm) 0.85 (s, 3H), 1.00 (s, 3H), 1.12 (t, J = 7.2 Hz, 3H), 1.95 (d, J = 16.0 Hz, 1H), 2.14 (d, J = 16.0 Hz, 1H), 2.22–2.30 (m, 4H), 2.40 (d, J = 16.8 Hz, 1H), 3.97 (q, J = 7.2 Hz, 2H), 4.74 (s, 1H), 6.54 (d, J = 8.4 Hz, 2H), 6.93 (d, J = 8.0 Hz, 2H), 8.94 (s, 1H), 9.01 (s, 1H); (Fig. S1); ^13^C NMR (126 MHz, DMSO) δ 194.3, 167.1, 155.3, 149.2, 144.4, 138.5, 128.4, 114.6, 114.4, 110.4, 104.1, 59.0, 34.8, 32.2, 29.2, 26.5, 18.3, 14.2 (Fig. S2).

#### Ethyl-4-(2-nitrophenyl)-2,7,7-trimethyl-5-oxo-1,4,5,6,7,8-hexahydroquinoline-3-carboxylate (4i)

^1^H NMR (500 MHz, DMSO): δ (ppm) 0.76 (s, 3H), 0.93–1.11 (m, 6H), 1.89 (d, J = 16.0 Hz, 1H), 2.15 (d, J = 16.0 Hz, 1H), 2.21—2.29 (m, 4H), 2.39 (d, J = 17.2 Hz, 1H), 3.82–3.96 (m, 2H), 5.65 (s, 1H), 7.29 (t, J = 8.0 Hz, 1H), 7.42 (d, J = 8.0 Hz, 1H), 7.55 (m, 1H), 7.72 (m, 1H), 9.12 (s, 1H); (Fig. S3). ^13^C NMR (126 MHz, DMSO) δ 194.0, 166.6, 149.8, 147.7, 146.0, 142.0, 130.7, 126.9, 123.7, 123.5, 109.9, 103.1, 59.1, 32.1, 28.9, 26.3, 18.3, 13.9 (Fig. S4).

#### Ethyl-4-(3-nitrophenyl)-2,7,7-trimethyl-5-oxo-1,4,5,6,7,8-hexahydroquinoline-3-carboxylate

^1^H NMR (500 MHz, DMSOd6): δ (ppm) 0.84 (s, 3H), 1.02 (s, 3H), 1.12 (t, J = 6.8 Hz, 3H), 1.97 (d, J = 16.0 Hz, 1H), 2.16 (d, J = 16.4 Hz, 1H), 2.27–2.35 (m, 4H), 2.46 (t, J = 16.8 Hz, 1H), 3.97 (q, J = 7.2 Hz, 2H), 4.97 (s, 1H), 7.50 (m, 1H), 7.61 (m, 1H), 7.97–7.99 (m, 2H), 9.24 (s, 1H); (Fig. S5). ^13^C NMR (126 MHz, DMSO) δ 194.4, 166.5, 166.4, 150.2, 149.8, 147.4, 146.4, 146.2, 134.47, 122.0, 120.9, 109.3, 102.7, 101.1, 59.3, 36.5, 32.2, 29.1, 26.3, 18.4, 14.0 (Fig. S6).

## Results and discussions

This study reports the synthesis of nanoporous Zn-MOF microspheres to develop an efficient and novel heterogeneous catalyst to synthesize polyhydroquinolines as an important dihydropyridine-containing scaffold via Hantzsch reaction. The resulting samples were characterized using various techniques.

### Synthesis and characterization of the catalyst

The heterogeneous Zn-MOF microsphere catalyst was successfully prepared by hydrothermal reaction of Zn(NO_3_)_2_·6H_2_O and 4,6-Diamino-2-pyrimidinethiol, using the deprotonation of amine and thiol groups. Afterwards, metal coordination bonding to the Zn ions was made to give a novel Zn-based microsphere catalyst. This newly synthesized nanoporous microsphere catalyst was fully characterized using various techniques; including, FT-IR, XRD, TGA, EDX, X-ray mapping, SEM, TEM and BET analyses. The results obtained from these techniques confirmed the successful preparation of this novel catalyst.

### Structural and chemical composition analysis

**Figure 1:**
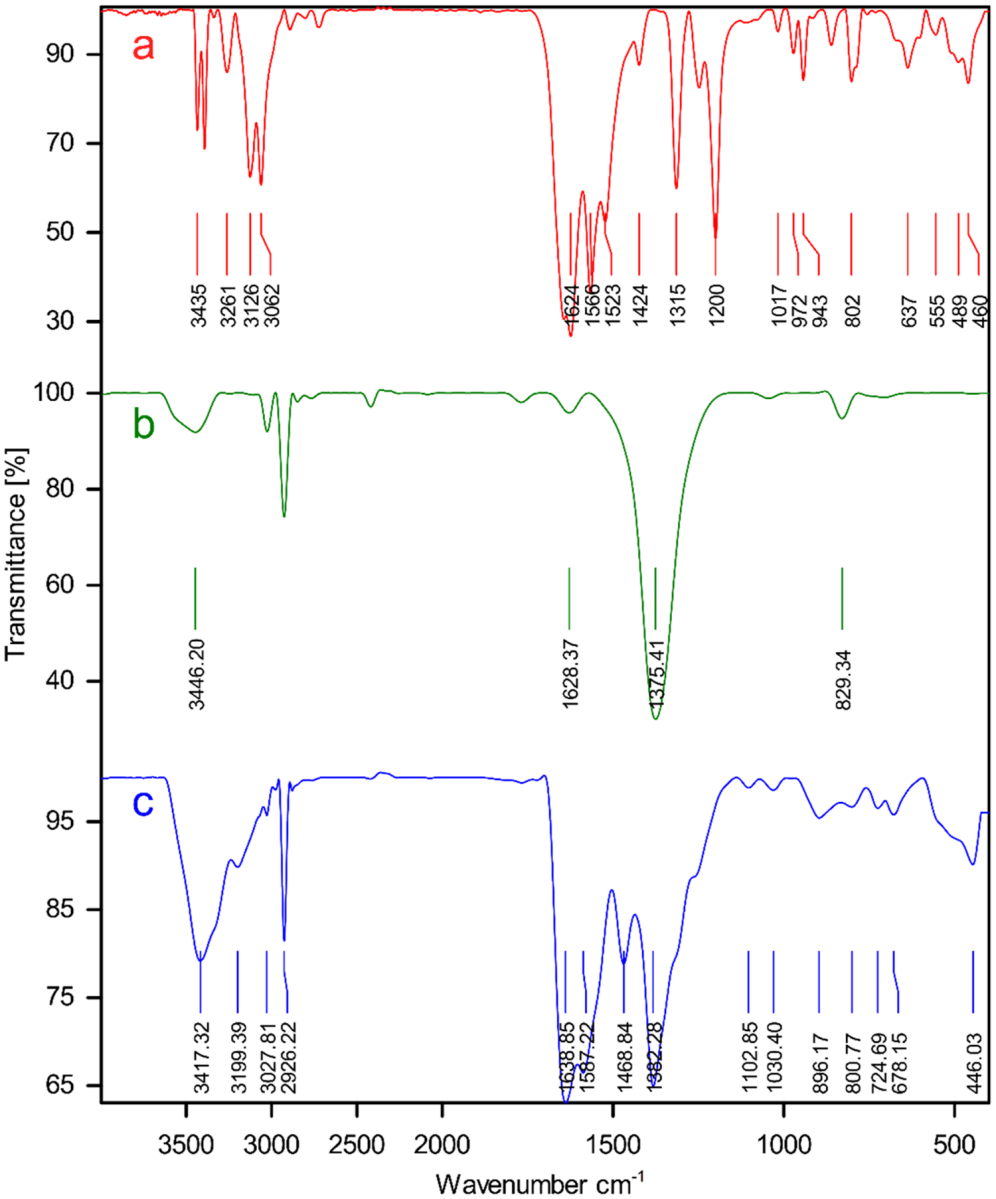
FT-IR Spectrums of (**a**) 4,6-Diamino-2-pyrimidinethiol, (**b**) Zn(NO_3_)_2_·6H_2_O and (**c**) nanoporous Zn-MOF microspheres.

The changes in the chemical features during the synthesis of the composite were measured by FT-IR spectroscopy. Figure 1 shows the FT-IR spectra of (a) 4,6-Diamino-2-pyrimidinethiol, (b) Zn(NO_3_)_2_.6H_2_O and (c) nanoporous Zn-MOF microspheres, respectively. Regarding the FT-IR spectrum of the 4,6-Diamino-2-pyrimidinethiol (Fig. 1a), the bands at 3339 cm^−1^ and 3435 cm^−1^ are attributed to the stretching vibration of N–H bonds of the free NH_2_ groups while those at 1523 cm^−1^ are related to the C=C group of the pyrimidine ring. The Amine bands disappeared in the FT-IR spectra of Zn-MOF microspheres (Fig. 1c), indicating that the NH_2_ groups have been deprotonated and coordinated to the Zn atoms. Moreover, the bending vibration of NH_2_ at 1638 cm^−1^ in the Zn-MOF microspheres shows a shift towards higher frequencies as compared to the primary sample. This shift can be assigned to the presence of Zn ions in MOF structure and the reduction of the single bond nature of the amine and thiol groups, which confirms the successful complexion of Zn ions with the N and S atoms in the framework. The strong C=C stretching vibration band at 1468 cm^−1^ and C-N stretch band at 1102 cm^−1^ provide shreds of evidence confirming the successful synthesis of the final MOF (Fig. 1c).

**Figure 2:**
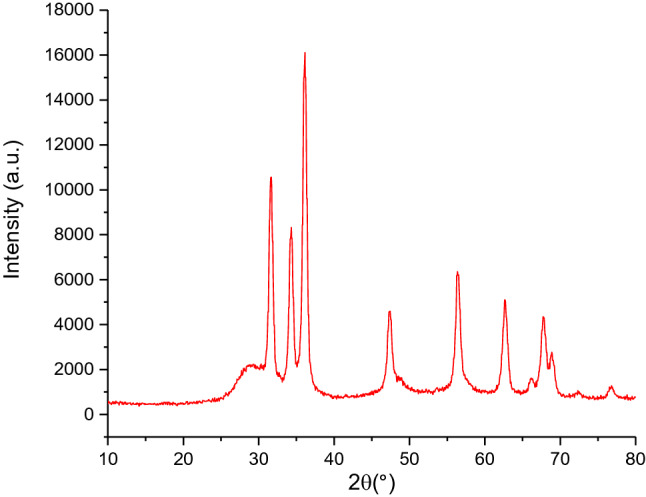
XRD pattern of nanoporous Zn-MOF microspheres.

The crystalline phases of nanoporous Zn-MOF microspheres were examined by Powder X-ray diffraction (PXRD) analysis as shown in Fig. 2. The characteristic diffraction peaks corresponding to the obtained framework are found at 2θ = 31.62°, 34.49°, 36.15°, 47.42°, 56.3°, 62.57°, 67.75°, 68.95°, 72.51° and 76.82° which effectively coincide with simulated XRD patterns, showing that the structure agrees with the literature reports^7,45,46^. Based on XRD results, we can also observe that the Zn-MOF microspheres obtained from zinc nitrate show sharp characteristic peaks, suggesting the high crystalline nature of the obtained nanoporous Zn-MOF microspheres.

**Figure 3:**
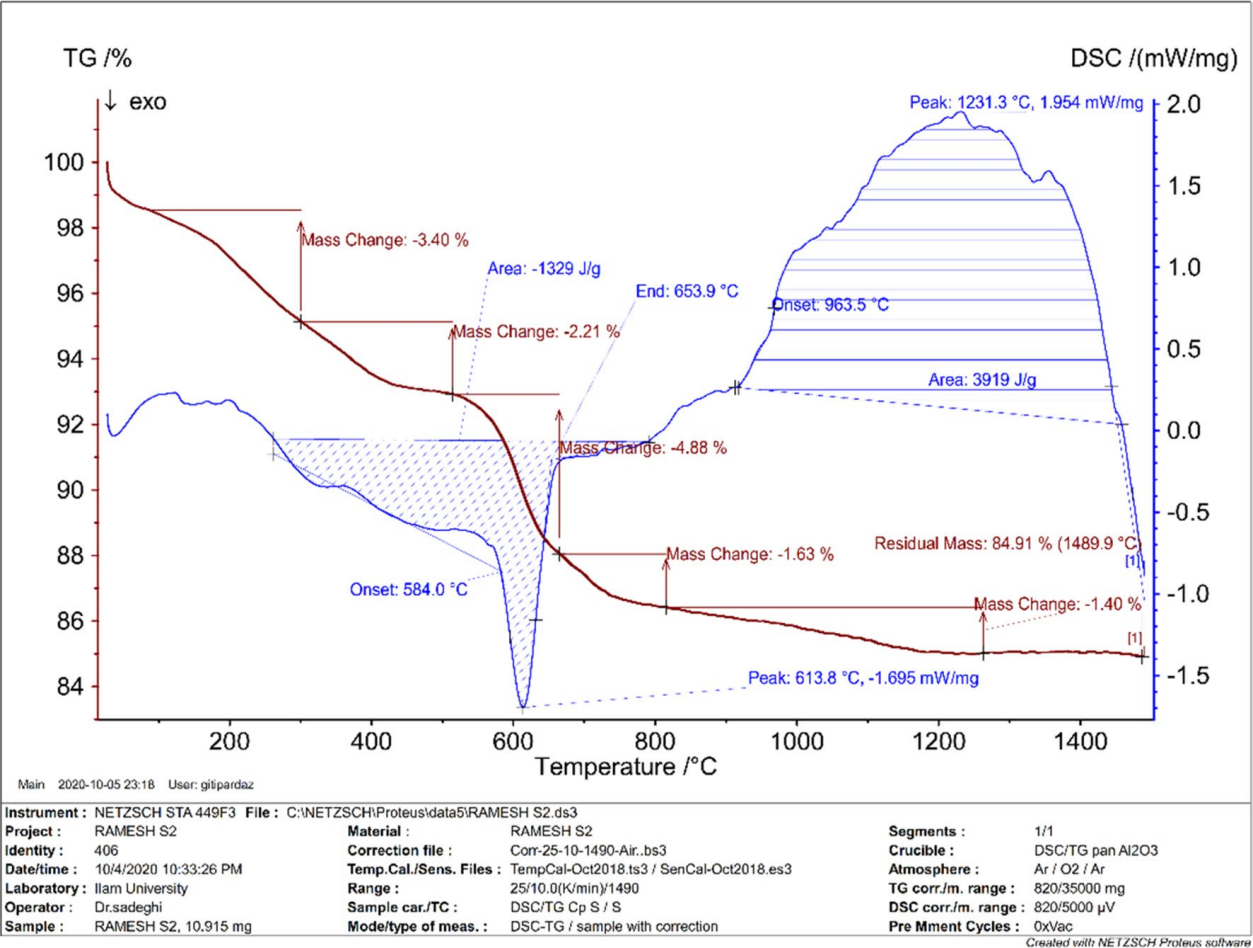
TGA (Maroon line) and DSC (Blue line) curves of nanoporous Zn-MOF microspheres.

To estimate the mass ratios and the thermal stability of nanoporous Zn-MOF microspheres, the TGA–DSC technique was employed. Figure 3 represents TGA (Maroon line) and DSC (Blue line) curves of Zn-MOF microspheres at the temperature range of 25–1500 °C. Regarding the TGA curve, the weight loss which was occurred below 200 °C was attributed to the release of the physically adsorbed moisture, water and organic solvents from the sample^47^. The next weight loss (10.61%) in the region of 200–800 °C can be associated with the decomposition of 4,6-Diamino-2-pyrimidinethiol ligand^48,49^. In addition, the weight loss at 450 °C indicates the decomposition of the framework. A basic point about the nature of the Zn-MOF catalyst is the thermal stability of the catalyst up to 200 °C. Therefore, this catalyst can be used under various catalytic reaction conditions up to 200 °C.

**Figure 4:**
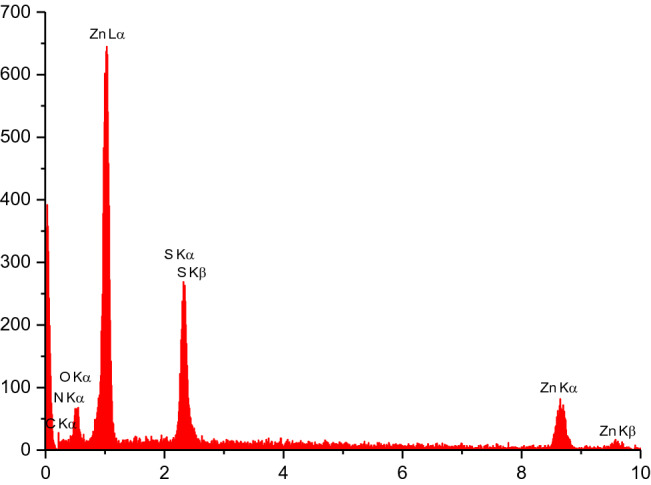
EDX Analysis of nanoporous Zn-MOF microspheres.

As shown in Fig. 4, energy dispersive X-ray (EDX) analysis was applied to determine the chemical composition of nanoporous Zn-MOF microspheres. The results indicate the presence of 53.25 W% of Zn species in the obtained nanoporous Zn-MOF microsphere catalyst. In addition, the presence of carbon (10.70 W%), nitrogen (9.11 W%), sulfur (13.97 W%) and oxygen (12.97 W%) elements in the prepared nanoporous material was also confirmed by these measurements. These observations support the high purity of the prepared Zn-MOF microspheres.

**Figure 5:**
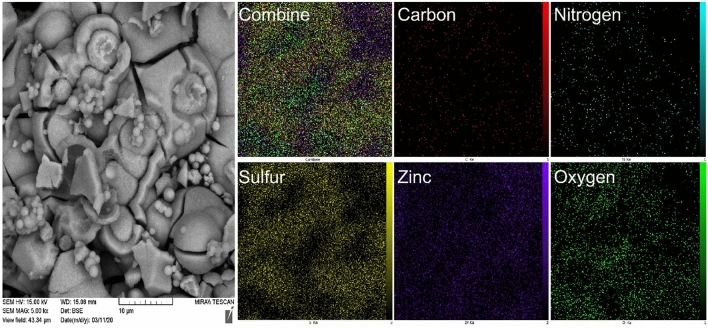
X-ray mapping analysis of Zn-CA-CPS.

The uniform distribution of the index elements (C, S, N, O, and Zn) of the obtained nanoporous material is observed at the X-ray mapping analysis (Fig. 5). In addition, the uniform distribution of the Zn element shows that it has evenly coordinated to N and S elements, indicating the presence of ligand in the obtained frameworks. Besides, the uniform incorporation of the activated Zn catalytic species with S and N groups shows that an excellent catalytic surface has been formed.

**Figure 6:**
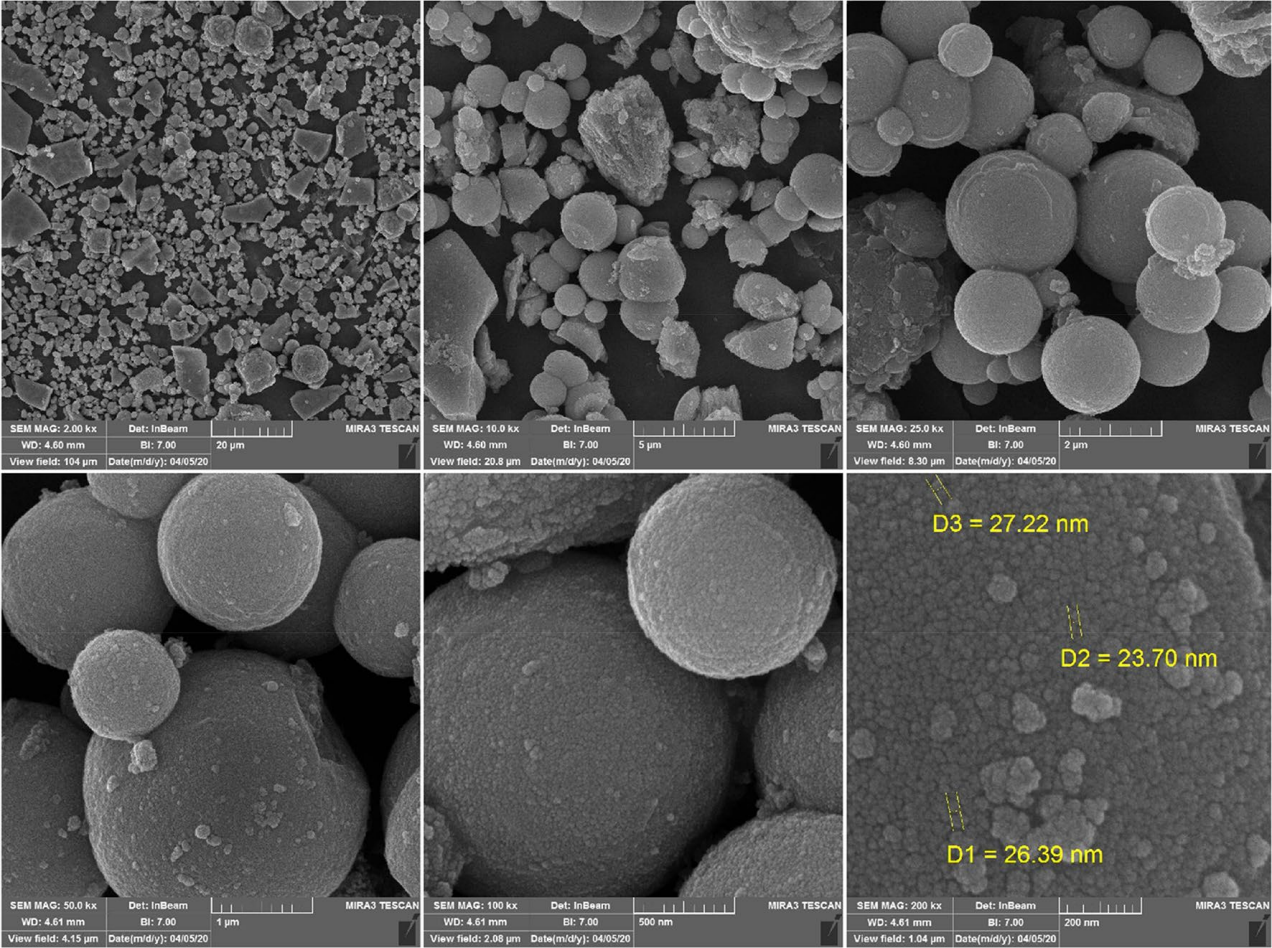
SEM images of three-dimensional walnut-like, hierarchically nanoporous Zn-MOF microspheres.

The surface morphology of the prepared nanoporous material was assessed by scanning electron microscopy (FE-SEM) and transmission electron microscopy (TEM) analyses. The FE-SEM images for pure nanoporous Zn-MOF microspheres are presented in Fig. 6. A closer look at the SEM images of the obtained nanoporous material indicates that the Zn-MOF has been formed in a uniform, three-dimensional, walnut-like and hierarchically nanoporous microparticle morphology, which can be attributed to a proper bonding in-between the metal and linker. Moreover, the SEM images show that these Zn-MOF microparticles are composed of a collection of nanoparticles having a mean particle size of 23–27 nm. The results indicate that the Zn-MOF microspheres have been formed in a uniform manner. Besides, there is no indication of agglomeration of the particles which results in optimizing the catalytic properties of the samples.

**Figure 7:**
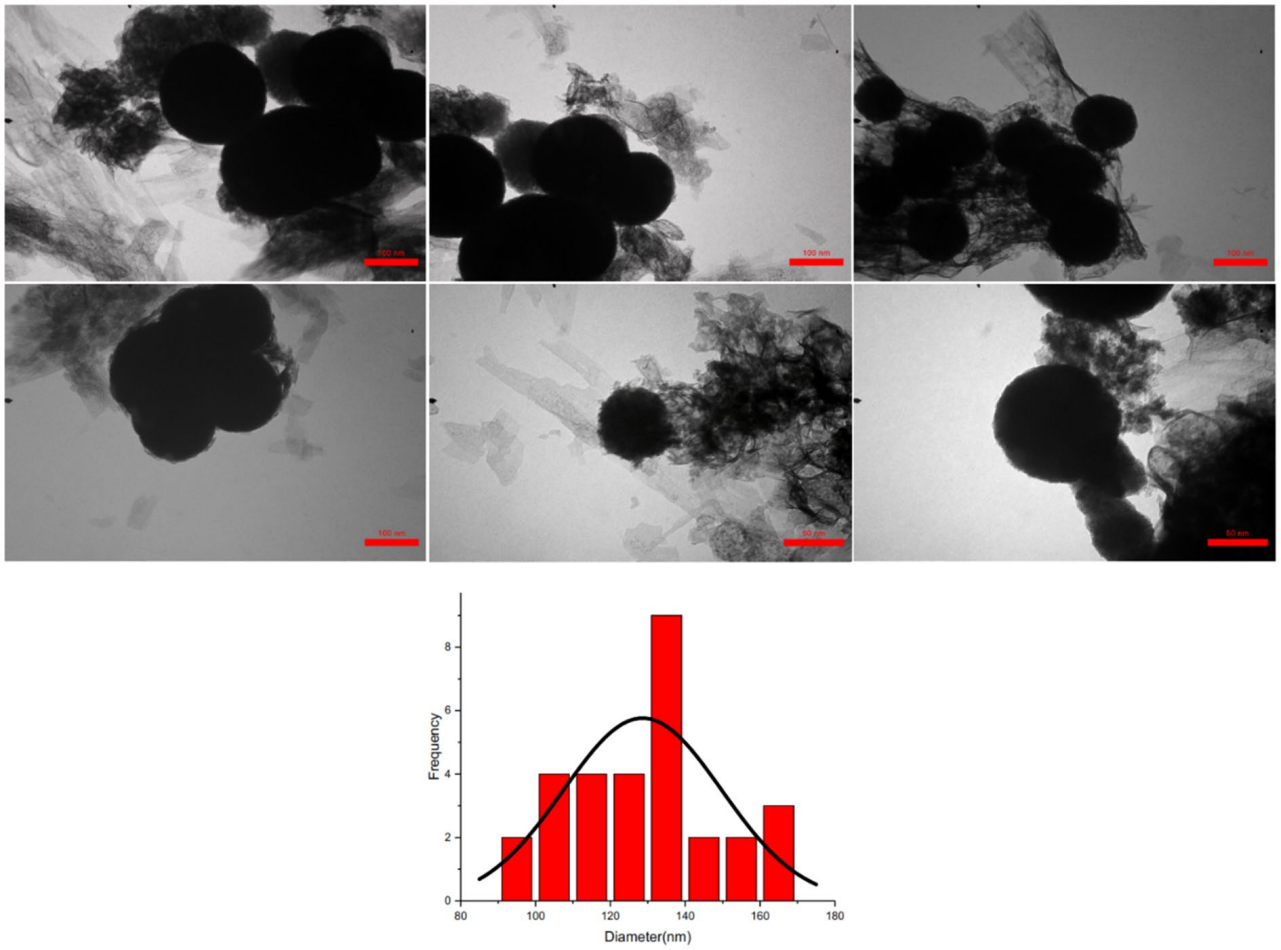
TEM images and particle size distribution histogram of Zn-MOF microspheres.

By applying the transmission electron microscopy technique, high quality images from the synthesized crystalline nanoporous Zn-MOF microspheres were obtained (Fig. 7). Evidently, the synthesized Zn-MOF microspheres showed characteristics of crystalline spherical structure, referring to the fact that the optioned material is a solid sphere. Particle size distribution histogram of the as-prepared microspheres gave distribution dimensions across a range from 90 to 170 nm, with an average size of 130 nm (Fig. 7).

**Figure 8:**
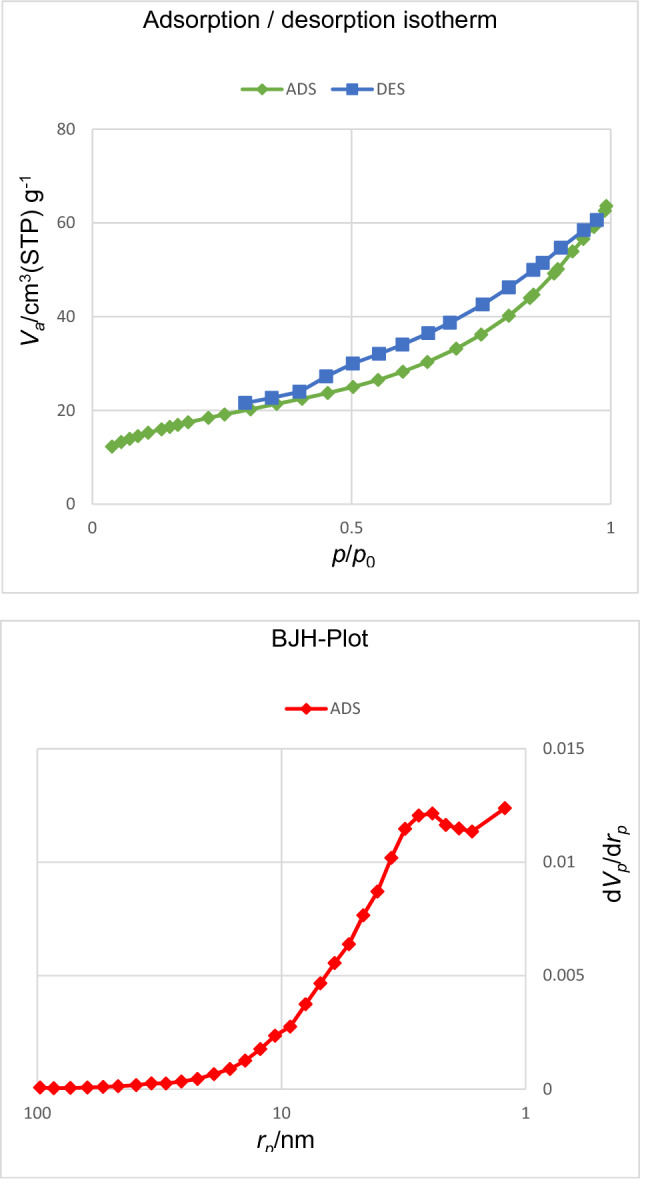
N_2_ adsorption/desorption isotherms and BJH-Plot of the nanoporous Zn-MOF microspheres.

The porosity and surface characteristics of nanoporous Zn-MOF microspheres were explored by the N_2_ adsorption/desorption analysis. The obtained results are illustrated in Fig. 8. Accordingly, Zn-MOF microspheres exhibited a Brunauer–Emmett–Teller (BET) surface area of 63.33 m^2^ g^−1^. Besides, the pore volumes and pore size distribution of nanoporous Zn-MOF microspheres are calculated by BJH analysis, as 0.088 cm^3^g^−1^ and 1.22 nm, belonging to the range of micropores materials.

## Catalytic study

After the successful characterization of the synthesized Zn-MOF microsphere, we applied these nanoporous materials as a novel catalyst for the multicomponent synthesis of polyhydroquinolines under diverse conditions (Table 1). Moreover, we optimized conditions of the reaction for Hantzsch synthesis of dihydropyridine scaffolds using *para*-chlorobenzaldehyde, dimedone, ethyl acetoacetate, and ammonium acetate as the model reaction. Afterwards, various parameters of the reaction, including the amount of catalyst, solvent and temperature, were evaluated for the model reaction, the results of which are summarized in Table 1. When there is no catalyst in the reaction, no reaction takes place (Table 1, entry 1). In this sense, it is worth mentioning that the existence of Zn-MOF microsphere is required for this type of Hantzsch reaction. The reaction proceeds faster by increasing the catalyst amount up to 10 mg. According to the results, 10 mg of the catalyst is required for the reaction. Besides, using smaller amounts of the catalyst will cause the reaction to be incomplete (Table 1, entry 4 & 5). Increasing the amount of the catalyst by more than 10 mg does not affect the efficiency percentage (Table 1, entry 7). Additionally, the catalytic effect of 4,6-diamino-2-pyrimidinethiol and Zn(NO_3_)_2_·6H_2_O was investigated on the model reaction. It was observed that they cannot efficiently catalyze the reaction and, as a result, the product is obtained in low yields in 85 min. Among various solvents used in the reaction, the results indicate that the PEG-400 which showed a higher efficiency, as compared to all solvents tested with 96% of isolated yield, may serve as both the solvent and the phase transfer catalyst in this type of reaction (Table 1, entry 6). Finally, the reaction was performed at different temperatures and, significantly, we observed that low temperature continued with lower efficiency (Table 1, entry 6, 12–14). Regarding the optimization studies, the optimum conditions for this reaction are 10 mg of Zn-MOF in the PEG-400 at 80 °C (Table 1, entry 6).

**Table 1:** Optimization of the reaction conditions for the Hantzsch condensation of *para*-Chlorobenzaldehyde, dimedone, ethyl acetoacetate, and ammonium acetate as the model reaction for the synthesis of polyhydroquinolines.


Entry	Catalyst	Amount Catalyst (mg)	Solvent	Temperature (°C)	Time (min)	Yield (%)^a,b^
1	–	–	PEG-400	80	85	30
2	4,6-Diamino-2-pyrimidinethiol	10	PEG-400	80	85	41
3	Zn(NO_3_)_2_.6H_2_O	10	PEG-400	80	85	30
4	Zn-MOF	5	PEG-400	80	85	75
5	Zn-MOF	6	PEG-400	80	85	79
6	Zn-MOF	10	PEG-400	80	85	96
7	Zn-MOF	12	PEG-400	80	85	92
8	Zn-MOF	10	EtOH	80	195	83
9	Zn-MOF	10	H_2_O	80	120	N.R
10	Zn-MOF	10	DMF	80	210	60
11	Zn-MOF	10	DMSO	80	95	70
12	Zn-MOF	10	PEG-400	25	120	N.R
13	Zn-MOF	10	PEG-400	60	85	21
14	Zn-MOF	10	PEG-400	100	85	44

^a^Isolated yield.

^b^Reaction conditions: 4-Chlorobenzaldehyde (1 mmol), dimedone (1 mmol), ethyl acetoacetate (1 mmol), ammonium acetate (1.2 mmol), catalyst (mg) and solvent (2 mL).

After optimizing the reaction conditions, we explored the scope of the reaction with various electron-donating and electron-withdrawing groups of aldehydes. In all cases, the products were made in high yields (Table 2). Although the results indicate that ortho and/or meta-substituted aryl aldehydes react slowly, as compared to the para isomers, electron-releasing and electron-withdrawing groups give an excellent yield of the product.

**Table 2:** Hantzsch synthesis of polyhydroquinoline derivatives in the presence of Zn-MOF in PEG-400 at 80 °C.


Entry	Aryl aldehyde	Product	Time (min)	Yield (%)^a,b^	Melting point	
					Measured	Literature
1			150	83	216–218	217–219^50^
2			85	96	237–240	234–237^50^
3			120	74	252–255	252–255^51^
4			70	58	247–250	245–247^51^
5			180	69	201–204	203–204^50^
6			150	79	263–265	246–248^50^
7			75	95	173–175	176–178^50^
8			90	85	217–219	216–218^50^
9			90	97	217–219	216–218^50^
10			150	92	227–229	231–233^51^
11			90	91	301–303	303–305^50^

^a^Isolated yields.

^b^Reaction conditions: Aromatic aldehyde (1 mmol), dimedone (1 mmol), ethyl acetoacetate (1 mmol), ammonium acetate (1.2 mmol), Zn-MOF (10 mg) and PEG-400 (2 mL) at 80 °C.

^c^Reaction conditions: Aromatic aldehyde (1 mmol), dimedone (2 mmol), ethyl acetoacetate (2 mmol), ammonium acetate (2.4 mmol), Zn-MOF (20 mg) and PEG-400 ( 4 mL) at 80 °C.

**Scheme 2:**
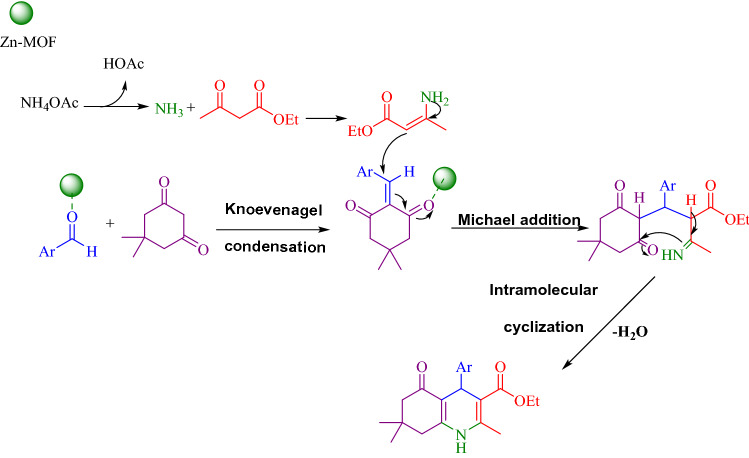
Proposed mechanism for the synthesis of polyhydroquinolines in the presence of nanoporous Zn-MOF microspheres.

It is worth noting that an appropriate mechanism for the synthesis reaction of polyhydroquinoline derivatives, catalyzed by nanoporous Zn-MOF microspheres, was proposed (Scheme 2)^36^. Firstly, the reaction was granted to start through deprotonation of the dimedone which underwent Knoevenagel condensation reaction with an aldehyde to form an α,β-unsaturated compound. Subsequently, the Michael addition of imine which was achieved from the combination of NH_3_ with ethyl acetoacetate on the α,β-unsaturated carbonyl compounds was followed by cyclization reaction, generating a six-membered ring. Finally, the dehydration gave the final polyhydroquinoline products (Scheme 2)^36^.

## Catalyst reusability studies

**Figure 9:**
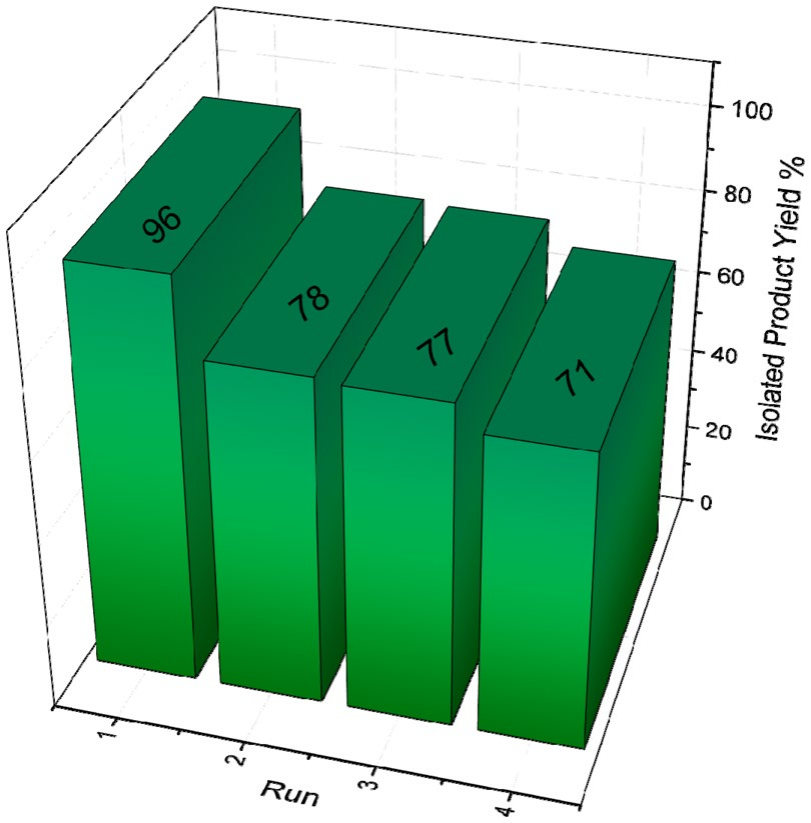
Recyclability of the nanoporous Zn-MOF microspheres.

**Figure 10:**
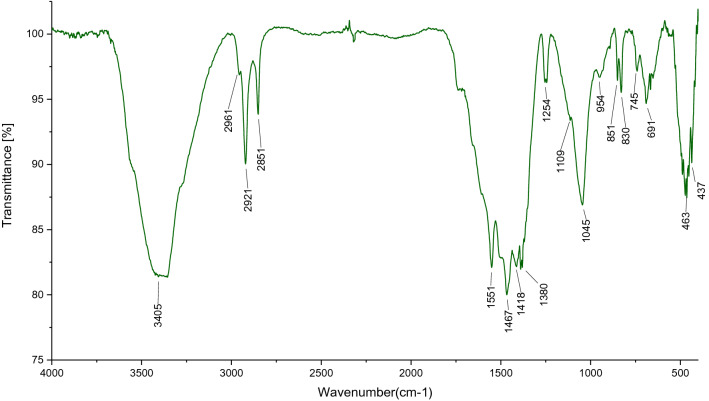
FT-IR Spectrum of spent nanoporous Zn-MOF catalyst.

Recyclability of the heterogeneous catalyst put an end to the use of harmful and costly metal catalysts while decreasing the cost of products. These factors are of crucial importance from an economical point of view. Recyclability of the nanoporous Zn-MOF microsphere was investigated in the model reaction. The catalyst was separated after completion of the reaction, washed with ethyl acetate and acetone and, then, dried at 70 °C. The dried catalyst was reused for the next cycle. Moreover, the recycled catalyst was employed in the four sequential cycles; the yield of the reaction was decreased moderately after the fourth run of the reaction, as illustrated in Fig. 9. Furthermore, we aimed at investigating the FT-IR spectrum of the reused microporous Zn-MOF catalyst (Fig. 10). Evidently, the stability of as-prepared catalyst is confirmed due to the fact that, almost all of the characteristic peaks are the same as the fresh catalyst.

## Hot filtration

The hot filtration test was another analysis to approve the heterogeneous nature of the nanoporous Zn-MOF microspheres in the Hantzsch synthesis of polyhydroquinolines. On this basis, the model reaction was studied again under the optimized reaction condition. After 43 min (59% conversion), the Zn-MOF was removed from the reaction by simple filtration. Afterwards, the rest of the reaction was stirred in the absence of the catalyst for a further 43 min. The obtained results show that the Zn-based MOF played a catalytic role in the reaction without the Zn leaching into the solution or framework degradation.

## Comparison

In the last part of our studies, to demonstrate the profit of nanoporous Zn-MOF microspheres as a heterogeneous catalyst in Hantzsch reaction, our resultant and reaction conditions were compared with those of the reported acid, base and metal catalysts in the synthesis of polyhydroquinolines (Table 3). As depicted in Table 3, the nanoporous Zn-MOF microspheres are the most efficient catalysts to synthesize polyhydroquinolines. Significantly, most of the reported methods toil from the absence of commonness for the condensation reactions of the deactivated aldehydes. In addition, the reported synthetic paths have some limitations, such as requiring extreme temperature or long duration, large amounts of the catalyst and, most importantly, the use of hazardous solvents to give excellent yields. Promising results obtained in the presence of Zn-MOF microspheres should be ascribed to the nanoporous structure of the catalyst. The two roles of PEG-400 (solvent and phase transfer catalyst) avouch the better contact between the catalyst and the reactants and, thus, substantially raising the catalytic activity and stability.

**Table 3:** Comparison of the polyhydroquinolines synthesis in presence of various catalysts.

Entry	Catalyst	Time (min)	Yield (%)^a^	Ref.
1	FeAl_2_O_4_	180	90	^52^
2	Fe_3_O_4_@D-NH-(CH_2_)_4_-SO_3_H	90	86	^53^
3	Fe_3_O_4_@FSM-16-SO_3_H	25	86	^54^
4	Fe_3_O_4_-TEDETA-Br_3_	120	92	^55^
5	Fe_3_O_4_@SiO_2_@(CH_2_)_3_Im}C(NO_2_)_3_	18	89	^56^
6	Fe_3_O_4_@SiO_2_–PEG/NH_2_	20	96	^57^
7	Fe_3_O_4_@GA@IG	45	89	^41^
8	AIL-SCMNPs	15	80	^58^
9	Zn-MOF	85	96	This work

^a^Isolated yield.

## Conclusion

In conclusion, we have reported an efficient and novel three-dimensional walnut-like, microporous Zn-MOF microsphere catalytic system by hydrothermal method. Significantly, we were able to accelerate the asymmetric Hantzsch reaction with various aryl aldehydes including electron-donor and electron-acceptor groups for in situ production of polyhydroquinolines. The present protocol gives an excellent yield of the product within a short reaction time, by an easy workup and no need for further purification of the product. The novelty, simple synthesis procedure, no use of harmful solvents, facile filtration, high yield and, especially, reusability are some of the advantages of the developed catalyst.

## Supplementary Information

Supplementary Information.
