# Supplementary material for: Microporous hierarchically Zn-MOF as an efficient catalyst for the Hantzsch synthesis of polyhydroquinolines
Source: Sci Rep. 2022 Jan 27;12:1479. doi: 10.1038/s41598-022-05411-8 (PMC8795191; doi:10.1038/s41598-022-05411-8)
Supplement: Supplementary file 1 — Supplementary Information. [file 41598_2022_5411_MOESM1_ESM.docx]

**Microporous Hierarchically Zn-MOF as an efficient Catalyst for the Hantzsch Synthesis of Polyhydroquinolines**

*Sayed Mohammad Ramish ^a^, Arash Ghorbani-Choghamarani ^b,*^, Masoud Mohammadi ^a^*

^a^ Department of Chemistry, Faculty of Science, Ilam University, Ilam, Iran.

^b^ Department of Organic Chemistry, Bu-Ali Sina University, 6517838683, Hamedan, Iran.

*Corresponding Author: E-mail addresses: [a.ghorbani@basu.ac.ir](mailto:a.ghorbani@basu.ac.ir) & [arashghch58@yahoo.com](mailto:arashghch58@yahoo.com) (A. Ghorbani-Choghamarani)

**2.4. Selected spectral data**

**Ethyl-4-(4-hydroxyphenyl)-2,7,7-trimethyl-5-oxo-1,4,5,6,7,8-hexahydroquinoline-3-carboxylate:** ^1^H NMR (500 MHz, DMSO-d6): δ (ppm) 0.85 (s, 3H), 1.00 (s, 3H), 1.12 (t, J = 7.2 Hz, 3H), 1.95 (d, J = 16.0 Hz, 1H), 2.14 (d, J = 16.0 Hz, 1H), 2.22 - 2.30 (m, 4H), 2.40 (d, J = 16.8 Hz, 1H), 3.97 (q, J = 7.2 Hz, 2H), 4.74 (s, 1H), 6.54 (d, J = 8.4 Hz, 2H), 6.93 (d, J = 8.0 Hz, 2H), 8.94 (s, 1H), 9.01 (s, 1H); (**Figure S1**); ^13^C NMR (126 MHz, DMSO) δ 194.3, 167.1, 155.3, 149.2, 144.4, 138.5, 128.4, 114.6, 114.4, 110.4, 104.1, 59.0, 34.8, 32.2, 29.2, 26.5, 18.3, 14.2 (**Figure S2**).

Ethyl-4-(2-nitrophenyl)-2,7,7-trimethyl-5-oxo-1,4,5,6,7,8-hexahydroquinoline-3-carboxylate (4i): ^1^H NMR (500 MHz, DMSO): δ (ppm) 0.76 (s, 3H), 0.93-1.11 (m, 6H), , 1.89 (d, J = 16.0 Hz, 1H), 2.15 (d, J = 16.0 Hz, 1H), 2.21 - 2.29 (m, 4H), 2.39 (d, J = 17.2 Hz, 1H), 3.82-3.96 (m, 2H), 5.65 (s, 1H), 7.29 (t, J = 8.0 Hz, 1H), 7.42 (d, J = 8.0 Hz, 1H), 7.55 (m, 1H), 7.72 (m, 1H), 9.12 (s, 1H); (**Figure S3**). ^13^C NMR (126 MHz, DMSO) δ 194.0, 166.6, 149.8, 147.7, 146.0, 142.0, 130.7, 126.9, 123.7, 123.5, 109.9, 103.1, 59.1, 32.1, 28.9, 26.3, 18.3, 13.9 (**Figure S4**).

**Ethyl-4-(3-nitrophenyl)-2,7,7-trimethyl-5-oxo-1,4,5,6,7,8-hexahydroquinoline-3-carboxylate**: ^1^H NMR (500 MHz, DMSOd6): δ (ppm) 0.84 (s, 3H), 1.02 (s, 3H), 1.12 (t, J = 6.8 Hz, 3H), 1.97 (d, J = 16.0 Hz, 1H), 2.16 (d, J = 16.4 Hz, 1H), 2.27 - 2.35 (m, 4H), 2.46 (t, J = 16.8 Hz, 1H), 3.97 (q, J = 7.2 Hz, 2H), 4.97 (s, 1H), 7.50 (m, 1H), 7.61 (m, 1H), 7.97-7.99 (m, 2H), 9.24 (s, 1H); (**Figure S5**). ^13^C NMR (126 MHz, DMSO) δ 194.4, 166.5, 166.4, 150.2, 149.8, 147.4, 146.4, 146.2, 134.47, 122.0, 120.9, 109.3, 102.7, 101.1, 59.3, 36.5, 32.2, 29.1, 26.3, 18.4, 14.0 (**Figure S6**).

**Figure S1**. ^1^H NMR (500 MHz, DMSO-d6) of: Ethyl-4-(4-hydroxyphenyl)-2,7,7-trimethyl-5-oxo-1,4,5,6,7,8-hexahydroquinoline-3-carboxylate.

**Figure S2**.^13^C NMR (126 MHz, DMSO-d6) of: Ethyl-4-(4-hydroxyphenyl)-2,7,7-trimethyl-5-oxo-1,4,5,6,7,8-hexahydroquinoline-3-carboxylate.

**Figure S3**. ^1^H NMR (500 MHz, DMSO) of ethyl-4-(3-nitrophenyl)-2,7,7-trimethyl-5-oxo-1,4,5,6,7,8-hexahydroquinoline-3-carboxylate.

**Figure S4.** ^13^C NMR (126 MHz, DMSO) of ethyl-4-(3-nitrophenyl)-2,7,7-trimethyl-5-oxo-1,4,5,6,7,8-hexahydroquinoline-3-carboxylate.

**Figure S5**. ^1^H NMR (500 MHz, DMSOd_6_) of ethyl-4-(3-nitrophenyl)-2,7,7-trimethyl-5-oxo-1,4,5,6,7,8-hexahydroquinoline-3-carboxylate

**Figure S6**. ^13^C NMR (126 MHz, DMSO) of ethyl-4-(3-nitrophenyl)-2,7,7-trimethyl-5-oxo-1,4,5,6,7,8-hexahydroquinoline-3-carboxylate.
